# Distinguishing tubal rupture from tubal abortion in ectopic pregnancies after methotrexate treatment: a retrospective cohort study

**DOI:** 10.1007/s00404-025-08069-5

**Published:** 2025-06-05

**Authors:** Asal Darwish, Sharon avishalom, Inshirah Sgayer, Susana Mustafa Mikhail, Lior Lowenstein, Ala Aiob

**Affiliations:** 1 Department of Obstetrics and Gynecology, Galilee Medical Center, POB 21, 22100 Nahariya, Israel; 2 Azrieli Faculty of Medicine, Bar Ilan University, Safed, Israel

**Keywords:** Tubal rupture, Tubal abortion, Transvaginal ultrasound, Free pelvic fluid, Beta-hCG, Surgical intervention, Predictive factors, Fallopian tube preservation

## Abstract

**Purpose:**

To identify clinical, sonographic, and laboratory characteristics that distinguish between tubal rupture and tubal abortion following methotrexate (MTX) treatment for ectopic pregnancy (EP) and to compare the morbidity associated with these 2 outcomes.

**Methods:**

This retrospective cohort study included women treated with MTX for EP at Galilee Medical Center between 2012 and 2024. Data on clinical presentation, ultrasound findings, and laboratory values were analyzed. Uregint surgical interventions were classified as tubal rupture or tubal abortion based on intraoperative findings. A comparative analysis between these groups was performed, and multivariable modeling was used to identify predictors of tubal rupture.

**Results:**

Among 280 women treated with MTX, 47 (16.7%) required urgent surgical intervention. Of these, 15 (34.9%) were confirmed as tubal rupture, while 28 (65.1%) were tubal abortion. Women with tubal rupture more frequently presented with free pelvic fluid on transvaginal ultrasound (64.3 vs. 28.6%, *P* = 0.045) and had significantly higher intraoperative blood loss (433 ± 143 mL vs. 250 ± 201 mL, *P* = 0.001). A multivariable logistic regression model identified free pelvic fluid as an independent predictor of tubal rupture (odds ratio: 6.09, 95% CI 1.23–30.09, *P* = 0.027). No significant differences in preoperative beta-hCG levels or other clinical symptoms were observed between the groups.

**Conclusion:**

Tubal rupture and tubal abortion share overlapping clinical features, making differentiation with current diagnostic tools challenging. Free pelvic fluid on ultrasound is a significant indicator of tubal rupture, underscoring the importance of timely surgical intervention. Recognizing that tubal abortion may be a self-limiting condition in some cases offers opportunities to preserve fallopian tube integrity and reduce unnecessary surgeries. Further research is needed to improve diagnostic accuracy and explore conservative management strategies for tubal abortion.

Date and number of trial registration: December 2024, 0138–24-NHR.

## What does this study adds to the clinical work

**Table:** 

Differentiating tubal rupture from tubal abortion in ectopic pregnancies is challenging. Free pelvic fluid on ultrasound strongly indicates rupture. However, over 65% of urgent surgeries for suspected rupture revealed tubal abortion instead, emphasizing the need for improved diagnostic accuracy.	

## Introduction

Ectopic pregnancy (EP), defined as the implantation of an embryo outside the uterine endometrium, occurs in approximately 1–2% of all pregnancies [1]. Advances in early detection, including transvaginal ultrasonography (TVS) and beta-human chorionic gonadotropin (beta-hCG) testing in the first trimester, have facilitated the timely diagnosis and management of EP [2]. Treatment options for hemodynamically stable patients include methotrexate (MTX) therapy or expectant management [3]. At the same time, surgery is required for those with failed MTX treatment, visible embryonic cardiac motion on ultrasound, recurrent EP in the same tube, suspected tubal rupture, or hemodynamic instability [4].

Single-dose MTX therapy (50 mg/m^2^) achieves a success rate of 65–95%, with treatment efficacy monitored through serial beta-hCG measurements on days 0, 4, and 7 [5–7]. A meta-analysis of 26 studies involving 1327 women reported an overall MTX success rate of 89% [7]. However, treatment failure occurs in 7–12% of cases, often associated with risk factors such as initial beta-hCG levels > 5000 mIU/mL, gestational sac size > 4 cm, fetal cardiac activity, or significant free peritoneal fluid on ultrasound [8, 9]. Tubal rupture, a severe complication of MTX failure, may occur even in patients without these risk factors [8, 10].

Despite extensive research, identifying specific risk factors for tubal rupture after MTX treatment remains challenging. Previous studies have examined risk factors for rupture in patients receiving MTX but did not distinguish between tubal rupture and tubal abortion [11, 12]. Additionally, moderate success has been achieved in predicting tubal rupture or abortion during follow-up, complicating clinical decision-making [2, 12, 13].

Patients with tubal abortion may exhibit symptoms resembling tubal rupture, such as abdominal pain or free peritoneal fluid on TVS. This diagnostic overlap makes it difficult to differentiate between patients with resolving symptoms, such as in tubal abortion, and those experiencing active rupture with significant bleeding. Consequently, surgical interventions tend to be performed in cases that might otherwise resolve spontaneously.

This study aimed to identify characteristics at ectopic diagnosis, during MTX treatment follow-up, and at the presentation that could distinguish between tubal rupture and tubal abortion after MTX treatment. Furthermore, we sought to compare the morbidity associated with these two outcomes.

## Materials and methods

This retrospective cohort study was conducted at the obstetrics and gynecology department of the Galilee medical center, Israel. It included women diagnosed with EP who received single-dose MTX as first-line treatment between 2012 and July 2024. The study was approved by the institutional review board (Helsinki committee) of Galilee medical center and the Israeli ministry of health (approval number 0138–24-NHR, December 2024).

EP diagnosis and management followed departmental protocol based on the American college of obstetricians and gynecologists (ACOG) practice bulletin [3]. Women with positive serum beta-hCG and no evidence of an intrauterine gestational sac were evaluated for EP using TVS and serial beta-hCG measurements. Eligibility criteria for MTX treatment included hemodynamic stability, reliable follow-up, a beta-hCG level < 5000 IU/L, gestational sac diameter ≤ 4 cm, and absence of embryonic cardiac motion on TVS. Depending on follow-up feasibility, eligible women received a single intramuscular MTX dose of 50 mg/m^2^ as outpatients or inpatients. Beta-hCG levels were monitored on days 0 (injection day), 4, and 7 post-MTX. A reduction of ≥ 15% in beta-hCG levels between days 4 and 7 was considered successful, with weekly monitoring until beta-hCG normalized. If beta-hCG declined by < 15%, a second MTX dose was administered, followed by weekly monitoring.

Women presenting during follow-up with symptoms suggestive of ruptured EP were referred for laparoscopy. The suspicion of tubal rupture was based on clinical presentation, including abdominal pain, nausea, vomiting, shoulder pain, dizziness, or hemodynamic instability. Physical examination findings and sonographic features, including free fluid and blood clots in the abdominal cavity, further supported this.

Eligible participants were identified by systematically reviewing the hospital’s electronic medical records and surgical databases. All cases coded as “ectopic pregnancy” and treated with MTX during the study period were screened. Inclusion criteria were: diagnosis of EP confirmed by TVS and/or serial beta-hCG measurements; receipt of at least 1 dose of MTX as first-line treatment; and complete medical records, including clinical, laboratory, sonographic, and surgical data when applicable. Patients were excluded if they received surgical treatment without prior MTX therapy, had missing essential data, or underwent elective surgery for treatment failure unrelated to suspected tubal rupture. Only cases that met all inclusion criteria were enrolled for analysis.

Data collected included demographic and medical history (e.g., age, gravidity, parity, gestational age, history of prior abortions or ectopic pregnancies), clinical presentation (e.g., severe abdominal pain, dizziness, vomiting, shoulder pain), findings from a physical examination (e.g., abdominal tenderness, peritoneal signs), ultrasound characteristics (e.g., presence of an ectopic mass, mean diameter, pelvic fluid), and laboratory values (e.g., pre- and post-surgical β-hCG levels and hemoglobin levels).

Surgical findings were classified as follows: tubal rupture was defined as rupture of the fallopian tube accompanied by active bleeding. In contrast, tubal abortion was defined as the passage of ectopic conception material from the fimbriae into the peritoneal cavity without damage or rupture of the fallopian tube. The volume of blood loss was quantified based on the amount collected via suction during the procedure.

### Statistical analysis

For the descriptive analysis, we described the categorical data using frequencies and percentages. Continuous variables with normal distributions were presented as means ± standard deviations. Median values and ranges were used for variables that did not meet the normal distribution assumption. For the inferential analysis, we compared categorical variables between the groups using the Chi-square test or Fisher’s exact test (when expectancy < 5). According to the variable distributions, we compared continuous variables between the groups using the independent t-test or Wilcoxon rank-sum test. If a normal distribution was found, the independent t-test was presented; a histogram determined the distribution shape. A multivariable linear model was adapted to examine the correlation between the TVS findings and EP tubal rupture. The dependent variable was tubal rupture. Sonographic findings, EP mass size, and free fluid by TVS were included as independent variables.

## Results

A total of 292 women received MTX treatment for ectopic pregnancy at Galilee Medical Center between 2012 and July 2024. Eight women were lost to follow-up**,** and 4 had cervical or cesarean scar pregnancies; these cases were excluded from the analysis.

Therefore, 280 women treated with MTX for tubal ectopic pregnancy were included in the final study cohort.

Of these, 233 women (83.2%) were successfully treated, with 200 women (71.4%) achieving success after a single dose of MTX and 33 women (11.7%) after 2 doses. Surgical intervention was required in 47 women (16.7%) following MTX treatment, with 44 cases (15.9%) occurring after 1 MTX dose and 3 cases (1.1%) after 2 doses.

**Fig. 1:**
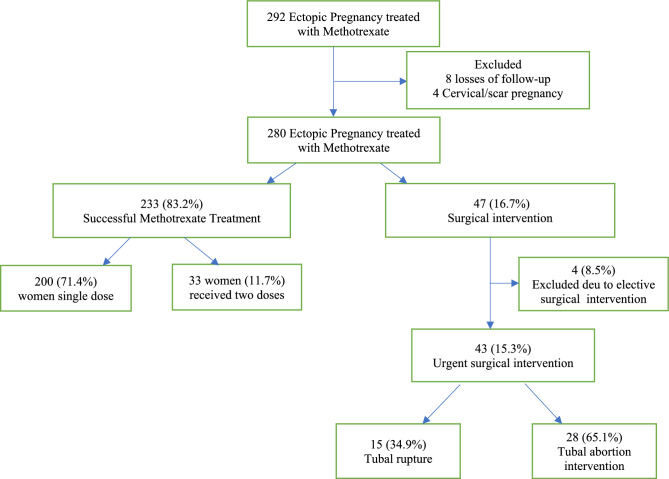
Treatment outcomes in women with ectopic pregnancy treated with methotrexate

Among the 47 women requiring surgery, 4 cases (8.5%) underwent elective surgery due to failed MTX treatment and were also excluded from the study. The remaining 43 (15.3%) women underwent urgent surgical intervention for suspected tubal rupture. Of these, 15 women (34.9%) were confirmed to have tubal rupture, while 28 women (65.1%) had tubal abortion (Fig. 1). A comparison between these 2 subgroups was performed.

The median time interval from MTX administration to urgent surgery was 2 days (2–23 days). Table 1 summarizes the 2 groups’ demographic, sonographic, and laboratory data. There were no significant differences in maternal age, history of prior abortions or ectopic pregnancies, or ectopic gestational age.

**Table 1:** Demographic, sonographic, laboratory, and operative findings of women with ectopic pregnancy treated with methotrexate who underwent urgent surgical intervention

N = 43	Tubal rupture (15) 34.9%	Tubal abortion (28) 65.1%	P value
Age, median (range)	34 (25–41)	31 (23–41)	0.444^*^
Abortion, median (range)	0 (0–2)	0 (0–3)	0.521^*^
Gravidity, median (range)	3 (1–6)	3 (1–6)	0.822^*^
Parity, median (range)	2 (0–4)	2 (0–5)	0.810^*^
EP in the past, (N)%	(1) 6.7%	(0)	0.349^**^
EP Gestational age at MTX treatment (weeks), median(range)	6.5 (5–9)	6 (5–9)	0.209^*^
Clinical findings			
Abdominal pain, (N)%	15 (100%)	22 (78%)	0.076^***^
Nausea and vomiting, (N)%	3 (20%)	4 (14.3%)	0.680^***^
Dizziness, (N)%	1 (6,7%)	1 (3.6%)	1.000^**^
Shoulder pain, (N)%	2 (13.3%)	1 (3.6%)	0.275^**^
Abdominal tenderness, (N)%	8 (53.3%)	10 (35.7%)	0.338^***^
Preoperative TVS findings			
EP by TVS- preoperative, (N)%	12 (85.7%)	23 (82.1%)	1.000^**^
EP size By TVS(cm), median (range)	2.3 (1–4)	1.5 (1–5)	0.097^*^ 2S 0.049^*^ 1S
Free fluid, (N)%	9 (64.3%)	8 (28.6%)	0.045^***^
Endometrium thickness(mm), median (range)	4 (2–15)	7 (5–25)	0.293^*^
Laboratory findings			
preoperative Bhcg, mean (± sd)	3337.6 (± 3168.3)	2166.6 (± 2327.4)	0.174^#^
Preoperative hemoglobin, mean (± sd)	11.4 (± 1.4)	11.8 ± 0.9	0.329^#^
Postoperative hemoglobin, mean (± sd)	10.1 (± 1.3)	10.5 ± 0.9	0.269^#^
Blood loss (cc), mean (± sd)	433 (± 143)	250 ± 201	0.001^#^

^*^ Mann–Whitney, ** Fisher’s, *** Chi-square, ^#^ t-test

Clinically, women with tubal abortion more frequently presented with abdominal pain compared to those with tubal rupture (100 vs. 78%, P = 0.076). However, no significant differences were observed in symptoms such as nausea, vomiting, dizziness, shoulder pain, or abdominal tenderness (Table 1).

Sonographically, no significant differences were found in the presence of an ectopic mass on TVS (85.7 vs. 82.1%, P = 1.000) or endometrial thickness (median 4 mm [2–15] vs. 7 mm [5–25], P = 0.293). However, free pelvic fluid was more common in the rupture group (64.3 vs. 28.6%, P = 0.045), and there was a marginally significant difference in ectopic pregnancy size (2.3 cm [1–4] vs. 1.5 cm [1–5], P = 0.097).

Biochemically, there were no significant differences in preoperative beta-hCG levels (3337.6 ± 3168.3 vs. 2166.6 ± 2327.4, P = 0.174) or hemoglobin levels before and after surgery (preoperative: 11.4 ± 1.4 vs. 11.8 ± 0.9, P = 0.329; postoperative: 10.1 ± 1.3 vs. 10.5 ± 0.9, P = 0.269). However, intraoperative blood loss was significantly higher in the tubal rupture group compared to the tubal abortion group (433 ± 143 mL vs. 250 ± 201 mL, P = 0.001). A multivariable linear model revealed that women with free fluid detected on transvaginal ultrasound at presentation were significantly more likely to be with tubal rupture rather than tubal abortion, with an odds ratio of 6.09 (95% CI 1.23–30.09, *P* = 0.027).

## Discussion

This study evaluates the clinical characteristics and outcomes of women who received MTX for EP and subsequently required urgent surgical intervention due to suspected tubal rupture. Our findings highlight critical distinctions between tubal rupture, a life-threatening condition, and tubal abortion, a potentially self-limiting condition, offering valuable insights to inform clinical decision-making and enhance management strategies.

In comparing the tubal abortion and tubal rupture groups, we observed a higher prevalence of free pelvic fluid on TVS in the rupture group (64.3 vs. 28.6%, P = 0.045), along with a marginally larger ectopic mass size (2.3 cm vs. 1.5 cm, P = 0.097) and a higher incidence of abdominal pain (100 vs. 78%, P = 0.076). Despite these findings, clinical symptoms such as nausea, dizziness, and shoulder pain were similar across both groups, with no statistically significant differences. These results highlight the challenge of differentiating between tubal abortion and tubal rupture. However, free fluid on TVS appears to have diagnostic value, as the likelihood of tubal rupture is 6 times higher in women with free fluid on TVS compared to those without, emphasizing the importance of sonographic evaluation in diagnosing rupture.

Intraoperative blood loss was significantly higher in the tubal rupture group (433 ± 143 mL vs. 250 ± 201 mL, P = 0.001). However, no significant differences in pre- or post-surgical hemoglobin levels were observed.

Our results are consistent with prior studies, which indicate that MTX treatment failure requiring urgent surgery can occur even without established risk factors, such as elevated beta-hCG levels, large gestational sac size, or embryonic cardiac activity [8, 10]. Our cohort’s overall MTX success rate was 83.2%, with 15.3% of patients requiring urgent surgical intervention, aligning with the 12–19% range reported in similar studies [5–7, 14, 15]. However, 65.1% of women who underwent surgery for suspected tubal rupture were ultimately diagnosed with tubal abortion, underscoring the diagnostic difficulty in distinguishing these 2 conditions based solely on clinical, sonographic, and laboratory findings. Notably, all women in this study underwent surgery for suspected tubal rupture within 23 days of receiving MTX, which is consistent with a recent study reporting EP rupture within 25 days following MTX treatment [16]. This interval is slightly longer than the typical 2-week window reported in earlier studies [12, 14]. This is, to our knowledge, a unique study to specifically differentiate between tubal rupture and tubal abortion in the context of MTX treatment. Prior studies often treated all surgical interventions for suspected rupture as cases of tubal rupture without distinguishing between rupture and abortion [2, 12, 17].

A review of the literature reveals a wide variation in the reported incidence of tubal rupture following MTX treatment, ranging from 20 to 77% [15, 18, 19]. This discrepancy is likely due to the interchangeable use of the terms “tubal rupture” and “severe abdominal pain requiring surgery.” It is essential, however, to distinguish between these 2 conditions, as severe abdominal pain is a common symptom that may indicate tubal abortion rather than true rupture. Our findings emphasize the clinical importance of this distinction: while tubal rupture is a life-threatening emergency requiring immediate surgical intervention, tubal abortion may in some cases resolve without surgery and my be maged conservatively. Supporting this, Lipscomb et al. reported that 79% of women hospitalized for persistent severe abdominal pain following MTX treatment did not ultimately require surgical intervention [20]. However, distinguishing between these conditions based on available clinical, sonographic, and laboratory data remains challenging.

Our findings have important clinical implications. Improved diagnostic tools, particularly more precise sonographic assessment of free pelvic fluid may enhance the ability to differentiate between tubal rupture and tubal abortion. In select, hemodynamically stable patients with non-conclusive signs of rupture, this distinction could support careful monitoring and potentially allow for conservative management, thereby reducing unnecessary surgical interventions and preserving fertility.

The significant number of women undergoing surgery for suspected tubal rupture who are ultimately diagnosed with tubal abortion highlights the necessity for improved diagnostic tools. Advanced imaging techniques and a better assessment of pelvic fluid characteristics, ectopic mass size, and beta-hCG trends may enhance diagnostic accuracy and reduce unnecessary surgeries. Future research should aim at developing predictive models or biomarkers to more accurately differentiate between tubal rupture and tubal abortion in women with suspected MTX treatment failure. Additionally, exploring conservative management approaches for tubal abortion could facilitate fallopian tube preservation, potentially improving long-term reproductive outcomes outcomes.

This study is the first to differentiate between tubal rupture and tubal abortion in the context of MTX treatment failure, addressing a significant gap in ectopic pregnancy management. It highlights the diagnostic value of free pelvic fluid on transvaginal sonography as a strong predictor of rupture, with tubal rupture being 6 times more likely when it is present.

However, the retrospective design and relatively small sample size limit the ability to draw causal inferences or generalize the findings to broader populations. Furthermore, the analysis was restricted to women who underwent urgent surgical intervention, excluding those with tubal abortion managed conservatively. As a result, we were unable to evaluate the rate or clinical outcomes of non-surgical resolution in such cases. Additionally, the study was limited by the lack of long-term reproductive outcome data and the absence of detailed sonographic characterization of free pelvic fluid.

## Conclusion

This study emphasizes the diagnostic challenge of distinguishing tubal rupture, which is a life-threatening emergency, from tubal abortion, which may resolve without surgical intervention. Free pelvic fluid on transvaginal sonography emerges as a critical diagnostic marker, highlighting the importance of sonographic evaluation in clinical decision-making. Future research should investigate conservative management strategies for tubal abortion to preserve fertility and improve long-term reproductive outcomes.

## Data Availability

No datasets were generated or analysed during the current study.
